# Rivaroxaban compared with low-dose aspirin in individuals with type 2 diabetes and high cardiovascular risk: a randomised trial to assess effects on endothelial function, platelet activation and vascular biomarkers

**DOI:** 10.1007/s00125-021-05562-9

**Published:** 2021-09-08

**Authors:** Frank Pistrosch, Jan B. Matschke, Dorothea Schipp, Bernhard Schipp, Elena Henkel, Ingo Weigmann, Jan Sradnick, Stefan R. Bornstein, Andreas L. Birkenfeld, Markolf Hanefeld

**Affiliations:** 1 Medical Clinic III, Universitätsklinikum ‘Carl Gustav Carus’, Dresden, Germany; 2 Independent Statistician, Rosenthal-Bielatal, Germany; 3 Faculty of Business and Economics, Department of Quantitative Methods, TU-Dresden, Dresden, Germany; 4 GWT TU-Dresden GmbH, Dresden, Germany; 5 Internal Medicine IV, Universitätsklinikum Tuebingen, Tuebingen, Germany; 6 Institute of Diabetes Research and Metabolic Diseases of the Helmholtz Centre Munich, Tuebingen, Germany

## Abstract

**Aims/hypothesis:**

Individuals with type 2 diabetes mellitus and subclinical inflammation have stimulated coagulation, activated platelets and endothelial dysfunction. Recent studies with the direct factor Xa inhibitor rivaroxaban in combination with low-dose aspirin demonstrated a significant reduction of major cardiovascular events, especially in individuals with type 2 diabetes and proven cardiovascular disease. Therefore, we asked the question of whether treatment with rivaroxaban could influence endothelial function, arterial stiffness and platelet activation.

**Methods:**

We conducted a multi-centre, prospective, randomised, open-label trial in 179 participants with type 2 diabetes (duration 2–20 years), subclinical inflammation (high-sensitivity C-reactive protein 2–10 mg/l) and at least two traits of the metabolic syndrome to compare the effects of the direct factor Xa inhibitor rivaroxaban (5 mg twice daily) vs aspirin (100 mg every day) on endothelial function (assessed by forearm occlusion plethysmography), skin blood flow (assessed by laser-Doppler fluxmetry), arterial stiffness (assessed by pulse wave velocity) and serum biomarkers of endothelial function and inflammation. Furthermore, we investigated phosphorylation of vasodilator-stimulated phosphoprotein (VASP) in platelets, the concentration of platelet-derived microparticles (PMPs) and the effects of isolated PMPs on HUVEC proliferation in vitro.

**Results:**

Rivaroxaban treatment for 20 weeks (*n* = 89) resulted in a significant improvement of post-ischaemic forearm blood flow (3.6 ± 4.7 vs 1.0 ± 5.2 ml/100 ml, *p* = 0.004), a numerically increased skin blood flow and reduced soluble P-Selectin plasma level vs aspirin. We did not find significant differences of arterial stiffness or further biomarkers. Neither rivaroxaban nor aspirin influenced VASP phosphorylation of platelets. The number of PMPs increased significantly with both rivaroxaban (365.2 ± 372.1 vs 237.4 ± 157.1 μl^−1^, *p* = 0.005) and aspirin (266.0 ± 212.7 vs 201.7 ± 162.7 μl^−1^, *p* = 0.021). PMPs of rivaroxaban-treated participants stimulated HUVEC proliferation in vitro compared with aspirin. Rivaroxaban was associated with a higher number of bleeding events.

**Conclusions/interpretation:**

Our findings indicate that the direct factor Xa inhibitor rivaroxaban improved endothelial function in participants with type 2 diabetes and subclinical inflammation but also increased the risk of bleeding.

**Trial registration:**

ClinicalTrials.gov NCT02164578.

**Funding:**

The study was supported by a research grant from Bayer Vital AG, Germany.

**Graphical abstract:**

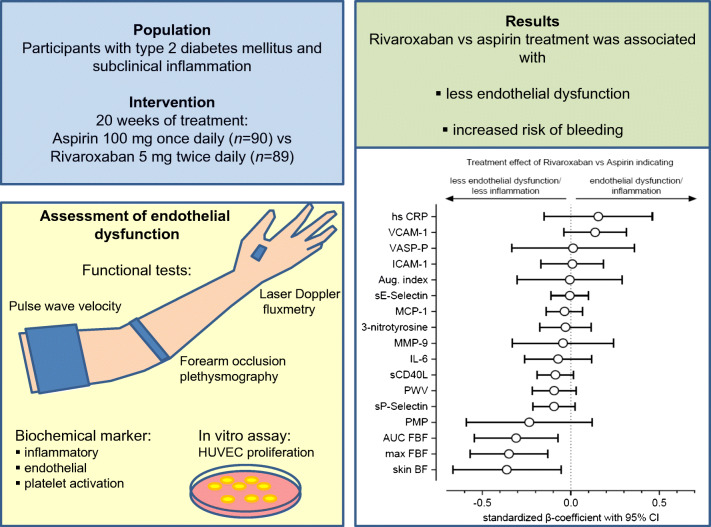

**Supplementary Information:**

The online version of this article (10.1007/s00125-021-05562-9) contains peer-reviewed but unedited supplementary material.

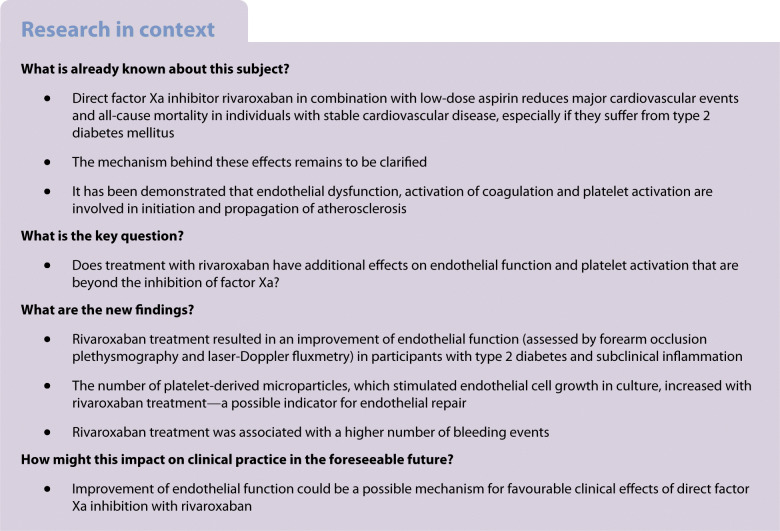



## Introduction

Type 2 diabetes mellitus with target organ damage or more than two additional risk factors has recently been classified as a very high-risk group for CVD [1]. There is increasing evidence that these individuals have subclinical inflammation, an activated coagulation pathway and activated platelets which can deteriorate microvascular blood flow and endothelial function with diminished release of endogenous vasodilators [2–4]. Endothelial dysfunction is most likely involved in both initiation and propagation of atherosclerosis and several studies suggest that measurement of endothelial function can be used as a proxy for cardiovascular risk [5].

Current treatment recommendations for secondary prevention of cardiovascular events in individuals with established macrovascular disease and diabetes mellitus include antiplatelet agents; however, this therapy has little or no effect for primary prevention of CVD [6]. A recent trial using the direct factor Xa inhibitor rivaroxaban in combination with aspirin, also known as acetylsalicylic acid, in individuals with stable CVD [7] demonstrated a significant reduction of major adverse cardiovascular events (MACE), especially in individuals with type 2 diabetes [8]. Rivaroxaban alone in this trial did not result in a net clinical benefit compared with aspirin mainly due to an increased number of bleeding events, but there was a superiority of rivaroxaban regarding the reduction of major adverse limb events in a subset of individuals with a history of peripheral artery disease [9]. The mechanism of this favourable effect remains to be clarified.

Animal and cell culture studies established a clear effect of factor Xa on platelet activation and endothelial cell dysfunction via the protease-activated receptors (PAR) [10]. Furthermore, there is some evidence from small clinical trials that beyond the anti-coagulatory action, inhibition of factor Xa could have additional anti-inflammatory actions that might evolve some beneficial effects in the microcirculation [11] or improve arterial stiffness, as observed in individuals with atrial fibrillation [12]. However, effects on endothelial function in individuals receiving direct factor Xa inhibition have never been measured before.

Therefore, we asked the question of whether treatment with the direct factor Xa inhibitor rivaroxaban in type 2 diabetic patients at high cardiovascular risk could influence endothelial function, microvascular blood flow, arterial stiffness, serum biomarkers of inflammation and platelet activation. The primary objective was the change of post-ischaemic forearm blood flow (FBF) during reactive hyperaemia (expressed as ∆FBF = FBF_hyperaemia_ − FBF_rest_) as a surrogate marker for endothelial function. Secondary objectives were change of skin blood flow during reactive hyperaemia as a marker of microcirculatory function, pulse wave velocity (PWV) as a marker of arterial stiffness, and assessment of different serum biomarkers of inflammation and endothelial function. An exploratory sub-study investigated platelet activation and the mitogenic capability of platelets in vitro.

## Methods

We conducted a multi-centre, prospective, randomised, open-label trial in type 2 diabetic patients with subclinical inflammation and hence endothelial dysfunction to compare the effects of the direct factor Xa inhibitor rivaroxaban vs aspirin (ClinicalTrials.gov registration no. NCT02164578). A complete list of investigators and study sites is provided in the Electronic supplementary material (ESM) Methods. Medical staff involved in the assessment of vascular function, analysis of laboratory biomarkers or in vitro analyses of samples were blinded to treatment arms and not involved in patient care.

**Fig. 1:**
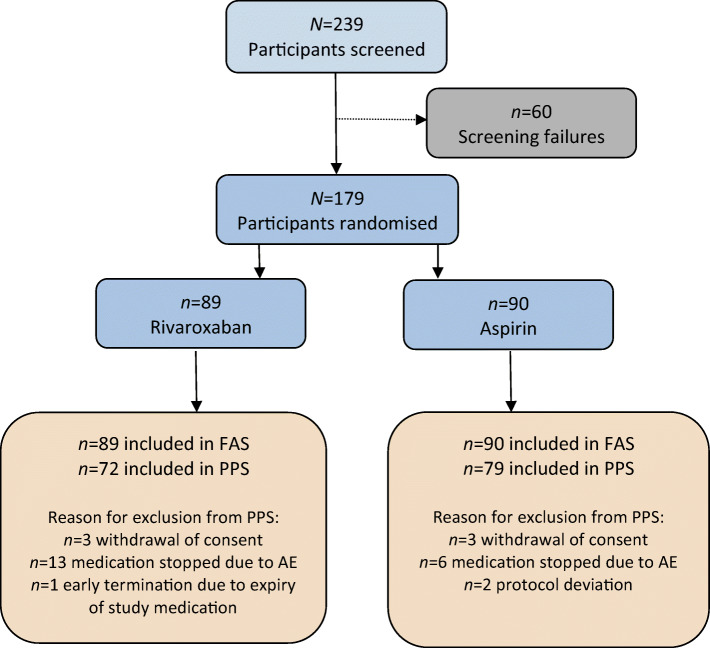
Flow chart of participant recruitment. FAS, full analysis set; PPS, per protocol set. AE, adverse event

We randomised 179 participants to receive rivaroxaban 5 mg twice daily (*n* = 89) or aspirin 100 mg once daily (*n* = 90) for 20 weeks. The flow diagram of participant recruitment is displayed in Fig. 1. The randomisation sequence generation and the time plan of recruitment are outlined in the ESM Methods. At baseline and end of treatment, measurements of vascular function were performed. Written informed consent was obtained from all participants prior to inclusion in the study. The study protocol has been approved by the ethical committee of the Saxony Chamber of Physicians, Dresden, Germany. The main inclusion criteria were type 2 diabetes mellitus with diabetes duration between 2 and 20 years, increased high-sensitivity C-reactive protein (hsCRP; an indicator of subclinical inflammation) and at least two traits of the metabolic syndrome or one of the following conditions: increased urinary albumin excretion, left ventricular hypertrophy or increased intima–media thickness of the common carotid artery. These inclusion criteria selected participants with a high probability of endothelial dysfunction and early arteriosclerosis. Furthermore, participants had to receive stable treatment with statins (if tolerated). Individuals with increased risk of bleeding or a proven cardiovascular event with indication for antiplatelet therapy were excluded from participation. A complete list of inclusion and exclusion criteria is given in the ESM Methods.

### Assessment of endothelial function

#### Venous occlusion plethysmography for assessment of FBF

Studies were performed in a quiet, temperature-controlled room and participants rested in a supine position for at least 20 min prior to the start of the examination. We used strain-gauge venous occlusion plethysmography (EC6 plethysmograph, Hokanson, WA, USA). A cuff for venous occlusion was placed at the upper arm. An elastic strain-gauge was placed on the maximal circumference of the forearm to assess changes of the circumference during 6 s of venous congestion at the upper arm. After assessment of baseline FBF (as the mean of five sequences of venous congestion at rest), forearm ischaemia was induced by inflating the upper arm cuff to 20 mmHg above the systolic value of the arterial blood pressure for 5 min. During forearm ischaemia, the release of endothelium-dependent vasodilators resulted in a maximal dilatation of forearm resistance vessels. The extent of endothelium-dependent vasodilatation was assessed after release of the upper arm cuff by sequences of venous occlusion. The velocity of forearm circumference increase during venous occlusion was highest immediately after release of forearm ischaemia (FBF_max_) and declined to the baseline value within 90 s. We assessed eight consecutive FBF values during this period (by automatic cycles of venous congestions) and calculated the area under the post-ischaemic FBF curve. Lower post-ischaemic FBF values indicate endothelial dysfunction. Venous occlusion plethysmography is highly reproducible (CV 1.8–2.9%) [13] and sensitive to detect short-term changes of endothelial function.

#### Laser-Doppler measurement of microcirculation

Microvascular skin blood flow was assessed using laser-Doppler fluxmetry (O2C, LEA Medizintechnik, Germany) as described [14]. The skin probe was placed at the dorsal thenar site of the left hand in between the phalanx of the thumb and metatarsal bone of the second digit. Measurements were performed at 2 mm depth with a continuously emitted laser light (wavelength 830 nm). The movement of erythrocytes within the sample volume causes a Doppler shift effect of the laser light which allows for the calculation of flow velocity and consequently the relative blood flow, which is expressed in arbitrary units. We measured the pre-ischaemic blood flow (pBF) and the maximal post-ischaemic blood flow (maxBF) during reactive hyperaemia after 5 min of suprasystolic ischaemia of the forearm. Mean pBF was calculated over 4 min at rest and maxBF was recorded as peak blood flow 20–40 s after cuff release. The same location of the skin probe was used for repeated measurements. Lower maxBF indicates reduced capillary recruitment and hence endothelial dysfunction. This method has lower reproducibility compared with venous occlusion plethysmography with a CV of 9.2% [15].

#### Serum biomarkers of endothelial function

We assessed established biochemical markers of endothelial function, platelet activation or endothelial–platelet interaction such as soluble P-Selectin (sP-Selectin), soluble E-Selectin (sE-Selectin), soluble vascular cell adhesion molecule 1 (VCAM-1) and soluble intercellular adhesion molecule 1 (ICAM-1). All analyses were performed using high-sensitivity ELISAs (IBL International, Germany) with high inter- and intra-assay reproducibility. The inter- and intra-assay CVs according to the manufacturer were 5.4% and 7.8% for sP-Selectin, <5% and <10% for sE-Selectin, 3.1% and 5.2% for VCAM-1, and 5.6% and 7.8% for ICAM-1, respectively. Cellular adhesion molecules mediate interactions among endothelial cells, platelets and mononuclear cells and are implicated in the progression of arteriosclerosis [16]. Furthermore, we assessed 3-nitrotyrosine by ELISA (Immundiagnostik AG, Germany; inter- and intra-assay CVs 2.8–5% and 8.8–12.3%, respectively). Nitrotyrosine arises from the nitration of free and protein-bound tyrosines; this mechanism is free radical dependent and a marker of oxidative stress [17]. Higher values of these biochemical markers indicate endothelial dysfunction.

### Assessment of arterial stiffness by PWV

Arterial stiffness is an accepted vascular biomarker and independent predictor of cardiovascular events and mortality. We measured PWV as the gold standard for assessment of arterial stiffness using an oscillometric device (MobilOGraph, IEM, Germany) with a cuff placed at the upper arm circumference. This technique provided information about PWV as well as the shape of the pulse wave and did not require a trained observer. It has been validated against tonometry as the current reference method of measurement [18]. Comparative studies demonstrated highly reproducible results during repeated measurements (CV 3.4%) [19].

### Biomarkers of inflammation

Blood samples were drawn at baseline and after 20 weeks of treatment for assessment of blood glucose level, cholesterol level, coagulation pathway and renal function. Furthermore, we assessed several biochemical markers of inflammation such as hsCRP, IL-6, monocyte chemoattractant protein 1 (MCP-1), matrix metallopeptidase 9 (MMP-9) and soluble CD40 ligand (sCD40L) using high-sensitivity ELISAs (IBL International) with inter- and intra-assay CVs between 4.0% and 7.8%, and 6.0% and 10.2%, respectively. Increased levels of inflammatory biomarkers are associated with atherosclerosis and endothelial dysfunction [20].

### Measures of platelet activation

In a subset of 105 participants (52 aspirin treated and 53 rivaroxaban treated) matched for age (±1 year), sex, diabetes duration (±2 years) and BMI (±1 kg/m^2^), we performed in vitro analyses of isolated platelets to assess phosphorylation of vasodilator-stimulated phosphoprotein (VASP), number of platelet-derived microparticles (PMPs) and effects of isolated PMPs on proliferation of HUVECs in vitro. HUVEC proliferation can be used as a model for endothelial repair. Human platelets, platelet rich plasma and platelet poor plasma were isolated at baseline and after 20 weeks of treatment as described [21] and stored at −80°C until use for further measurements.

VASP is an intracellular actin-binding protein which can be activated by phosphorylation via protein kinases A and G at three different phosphorylation sites: Ser_157_, Ser_239_ and Thr_278_. Phosphorylation of VASP within platelets can be stimulated by prostacyclins or nitric oxide (NO) and causes a stabilisation of platelet cytoskeleton, inhibition of shape change and hence a reduced platelet aggregation [22]. For assessment of Ser_157_ phosphorylation, isolated platelets were labelled with an allophycocyanin-conjugated CD41/61 antibody (Miltenyi Biotec, Germany) and a FITC-conjugated anti-phospho VASP Ser_157_ antibody (Nanotools, Germany) or a FITC-conjugated IgG1κ isotype control (Miltenyi Biotec) and analysed in a flow cytometer (BD-FACSCanto II, Becton Dickinson, CA, USA) using FlowJo version 6.5 software (Becton Dickinson).

Microparticles are circulating membrane fragments which contain surface markers of their original cells, can interact with the endothelium and are involved in endothelial repair [23]. Platelets are the main source of microparticles. Platelet poor plasma was used for isolation of PMPs. For quantification, PMPs were labelled with phycoerythrin (PE)-conjugated CD42b antibody or PE-conjugated recombinant engineered control antibodies (both Miltenyi Biotec) and analysed in a flow cytometer. PMPs were isolated using a magnetic particle concentrator (Thermo Fisher Scientific, WA, USA) after incubation with a biotin-labelled CD42 antibody (Miltenyi Biotec) and biotin-binding magnetic particles. Methodological studies reported an acceptable reproducibility for PMP isolation, with an intra-instrument CV between 2% and 11% [24]. We seeded 3000 HUVECs using a 96-well plate and endothelial cell growth medium (EGM-2, Lonza Bioscience, Switzerland) as nutrition solution. Via magnetic nanoparticles, isolated PMPs were incubated with the HUVECs for 72 h at 37°C. Thereafter, 4-[3-(4-iodophenyl)-2-(4-nitrophenyl)-2H-5-tetrazolio]-1,3-benzene disulfonate (WST-1 reagent, Sigma Aldrich, Germany) was added and cells were incubated for another 4 h. WST-1 is metabolised in the mitochondria via the NAD-dependant dehydrogenase into formazan. Formazan is reliable for the change of colour. We evaluated the formazan absorbance at 450 nm in combination with the reference at 595 nm using a photometer by Roche. In every measurement an empty control was measured as well [25]. Only cell suspensions characterised by endothelial cell purity ≥ 98% and low levels of apoptosis, evaluated by Annexin-V/propidium iodide double staining, were used for the in vitro assays. HUVECs were isolated from freshly obtained human umbilical cords which were donated after written informed consent of the mother.

### Statistical analysis

Statistical analysis was performed using IBM SPSS Statistics version 25 (www.ibm.com/de). All hypotheses were tested at a 5% level of significance against two-sided alternative hypotheses. In the case of normally distributed data, comparisons between groups were assessed by an ANCOVA procedure, which includes treatment as the main factor and baseline value, sex, age and disease duration as covariates. However, the model revealed only baseline value as a significant covariate. Non-normally distributed variables were log-transformed and the ANCOVA procedure was performed as described above if the hypothesis of normally distributed data had not been rejected. Otherwise, we used Mann–Whitney *U* test. Differences to baseline were analysed by a paired *t* test or Wilcoxon’s signed rank test. For testing of categorical variables, we used χ^2^ test or Fisher’s exact test. Furthermore, we performed a linear regression analysis for calculation of standardised β-coefficients of the treatment effect for all outcome variables. The corresponding ANCOVA uses treatment (not standardised), baseline and follow-up (both standardised, i.e. subtraction of the arithmetic mean and division by SD). A positive value of β demonstrates an increase of the corresponding variable by rivaroxaban treatment (vs aspirin). Descriptive data are represented as mean ± SD. A detailed description of statistical analyses is outlined in the ESM Methods.

## Results

Clinical characteristics of participants are demonstrated in Table 1. At baseline there were no significant differences regarding age, sex or diabetes duration and routine clinical laboratory markers. As expected, we found significant treatment effects of rivaroxaban on variables of coagulation (prothrombin time, activated partial thromboplastin time and prothrombin fragment [PTF] 1+2) which indicated a factor Xa inhibition and reduced thrombin formation. These changes of coagulation variables indirectly demonstrated compliance of participants with study medication intake.

**Table 1:** Clinical characteristics of participants at baseline and follow-up

Variable	Rivaroxaban (*n*=89)		Aspirin (*n*=90)		Comparison of groups
	Baseline	Week 20	Baseline	Week 20	
Age (years)	64.2 ± 7.4		64.7 ± 6.8		
Sex (m/f)	38/51		46/44		
Diabetes duration (years)	9.2 ± 5.1		9.2 ± 5.2		
BMI (kg/m^2^)	33.2 ± 5.3		33.4 ± 5.1		
Syst BP (mmHg)	134 ± 13	133 ± 13	132 ± 13	134 ± 13	−2.12 (−5.65, 1.42)
Diast BP (mmHg)	83 ± 9	81 ± 8	83 ± 10	82 ± 9	−1.21 (−3.47, 1.04)
HbA_1c_ (mmol/mol)	51.4 ± 6.0	51.4 ± 6.8	52.4 ± 7.5	51.0 ± 5.6	0.664
HbA_1c_ (%)	6.9 ± 0.8	6.9 ± 0.9	6.9 ± 1	6.8 ± 0.8	
LDL-C (mmol/l)	3.3 ± 0.9	3.5 ± 0.9^†^	3.3 ± 1.0	3.4 ± 0.8	0.2 (0.01, 0.38)
eGFR (ml/min)	98.8 ± 24.2	98.8 ± 25	95.5 ± 23.3	95.5 ± 23.1	0.944
UACR (mg/mmol)	2.9 ± 5	3.0 ± 6.5	3.8 ± 7.4	3.1 ± 7.3	0.817
Prothrombin time (%)	103.7 ± 9.5	81.4 ± 17.6*	101.4 ± 12.1	103.5 ± 11.4	<0.001
aPTT (s)	29.5 ± 2.02	33.2 ± 3.9^†^	29.7 ± 2.4	29.5 ± 2	3.92 (3.15, 4.69)
PTF 1+ 2 (pmol/l)	204 ± 89.9	173.2 ± 256.8*	211.3 ± 233.2	206.5 ± 106	<0.001
T/AT III (ng/ml)	6 ± 8.2	5.3 ± 8.3	9.5 ± 12.1	6.7 ± 10.1	0.743
D-dimer (ng/ml)	413.6 ± 212.6	418.5 ± 455.3*	412.8 ± 243.8	415.1 ± 236.2	0.066
von Willebrand factor (%)	152.2 ± 57.9	151.9 ± 56.5	148.4 ± 55.9	147 ± 54.8	0.710
Fibrinogen (g/l)	3.5 ± 0.7	3.7 ± 0.9	3.7 ± 0.7	3.6 ± 0.7	0.05 (0, 0.09)^a^
Protein C (%)	124.1 ± 20.5	123.6 ± 22.7	119.8 ± 21.4	118.2 ± 19.6	0.711
Protein S (%)	113.7 ± 22.5	110.8 ± 25.1*	109.7 ± 21.5	107.8 ± 17.8	0.120
Hematocrit (%)	41.1 ± 3.3	40.6 ± 3.0	41.2 ± 3.5	41.1 ± 3.5	0.02 (−0.03, 0.06)^a^

Comparison between groups (based on intraindividual differences between week 20 and baseline): effect size and 95% CI are given for ANCOVA or *p* values for Mann–Whitney *U* test

^a^Test on log-transformed data

Comparison vs baseline: *<0.02 (Wilcoxon’s signed rank test); ^†^<0.02 (paired *t* test)

aPTT, activated partial thromboplastin time; diast BP, diastolic BP; LDL-C, LDL-cholesterol; m/f, male/female; syst BP, systolic BP; T/AT, thrombin/antithrombin III complex; UACR, urinary albumin/creatinine ratio

**Fig. 2:**
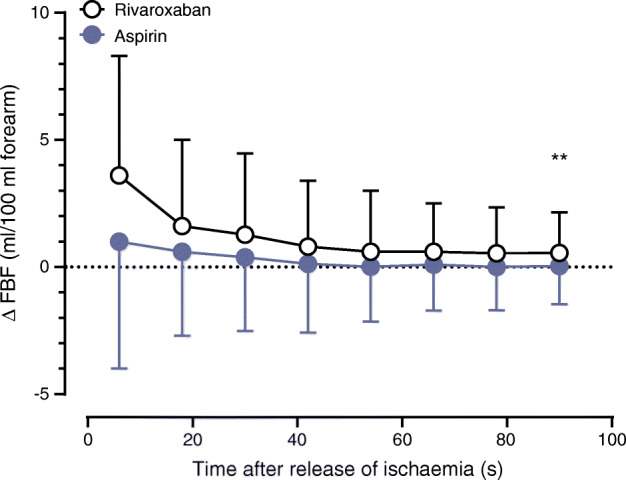
Post-ischaemic FBF (difference between week 20 and baseline); ANCOVA for repeated measures with resting blood flow as covariate. **p < 0.01

The primary endpoint of this study is outlined in Fig. 2. Rivaroxaban treatment for 20 weeks significantly increased post-ischaemic ∆FBF (eight consecutive measurements of post-ischaemic blood flow, analysed by ANCOVA for repeated measurements with resting FBF as covariate and first order autoregressive covariance structure) compared with aspirin treatment. The difference of maximal FBF (20 weeks of treatment–baseline) was 3.6 ± 4.7 vs 1.0 ± 5.2 ml/100 ml for rivaroxaban or aspirin, respectively (*p* = 0.004, Fig. 2). This finding indicates an improvement of endothelial function. We found both a higher maximal FBF as well as higher AUC of FBF after rivaroxaban (Table 2). FBF at rest was not significantly different between groups at baseline (2.5 ± 1 vs 2.6 ± 1.1 ml/100 ml) and after 20 weeks of treatment (2.3 ± 1 vs 2.7 ± 1.2 ml/100 ml for rivaroxaban or aspirin, respectively). Further indicators for improved endothelial function after rivaroxaban treatment were the numerically increased microvascular skin blood flow after forearm ischaemia (Table 2) and the reduction of sP-Selectin plasma levels compared with baseline. Effects on established vascular and inflammatory biomarkers are displayed in Table 2. Aspirin treatment resulted in a slightly but statistically significant decrease of hsCRP. PWV and pulse waveform analysis, expressed as augmentation index, did not differ significantly between groups (Table 2). We did not find any changes of VASP phosphorylation at Ser_157_ during treatment but there was a significant increase of circulating PMPs with both treatments compared with baseline (Table 2). Isolated PMPs of participants with rivaroxaban treatment increased HUVEC proliferation rate by 21% compared with baseline and aspirin treatment in vitro (ESM Fig. 1).

**Table 2:** Measures of endothelial function, arterial stiffness and inflammatory biomarkers

Variable	Rivaroxaban (*n*=89)		Aspirin (*n*=90)		Comparison of groups
	Baseline	Week 20	Baseline	Week 20	
Endothelial function					
AUC of FBF (ml/100 ml × s)	355 ± 227	445 ± 233*	348 ± 197	369 ± 225	0.021
Maximal ∆ FBF (ml/100 ml)	12.4 ± 5.6	16.0 ± 6.1*	13.5 ± 6.7	14.5 ± 7.1	0.001
∆ Skin blood flow (U)	58.3 ± 36.1	62.3 ± 32.7	57.3 ± 30	50.8 ± 27.7	0.629
VCAM-1 (pg/ml)	716 ± 225	740 ± 243	799 ± 419	807 ± 382	0.371
ICAM-1 (pg/ml)	296 ± 84	298 ± 85	285 ± 81	287 ± 84	0.00 (−0.04, 0.05)^a^
sE-Selectin (ng/ml)	87.2 ± 53	86.2 ± 52	81.9 ± 40	81.1 ± 39	−0.02 (−0.08, 0.04)^a^
sP-Selectin (ng/ml)	109.8 ± 37	102.9 ± 37*	114.5 ± 41	112.2 ± 43	0.116
3-Nitrotyrosine (pmol/ml)	294.2 ± 296	305.2 ± 338	319.7 ± 443	356.3 ± 627	0.383
Arterial stiffness					
PWV (m/s)	9.3 ± 1.2	9.3 ± 1.3	9.3 ± 1.1	9.4 ± 1.2	−0.11 (−0.26, 0.03)
Augmentation index (%)	26.9 ± 15.7	26.5 ± 16.8	28.6 ± 17.9	27.3 ± 16.3	0.985
Inflammation					
hsCrP (mg/l)	4.2 ± 3.2	5 ± 5.6	4.7 ± 3.8	4.6 ± 6*	0.25 (0.01, 0.48)^a^
MCP-1 (pg/ml)	546 ± 264	560 ± 338	523 ± 344	542 ± 402	0.297
IL-6 (pg/ml)	3.0 ± 6.2	3.5 ± 6.5	2.6 ± 5.4	3.5 ± 7.4	0.641
MMP-9 (pg/ml)	109.5 ± 74	115.1 ± 65	111.9 ± 66	122.5 ± 66	−0.04 (−0.2, 0.12)^a^
sCD40L (ng/ml)	3.2 ± 3.4	3.1 ± 3.3	4.0 ± 6.3	4.5 ± 8.1	0.141
Platelet activation					
VASP-P (%)	11.4 ± 9.8	11.6 ± 8.8	11.1 ± 9.8	11.7 ± 7.5	0.577
PMP (1/μl)	237.4 ± 157.1	365.2 ± 222*	201.7 ± 162.7	266 ± 212.7*	0.628

Comparison between groups (based on intraindividual differences between week 20 and baseline): effect size and 95% CI are given for ANCOVA or *p* values for Mann–Whitney *U* test

^a^Test on log-transformed data

Comparison vs baseline: *<0.02 (Wilcoxon’s signed rank test)

VASP-P, phosphorylated VASP

**Fig. 3:**
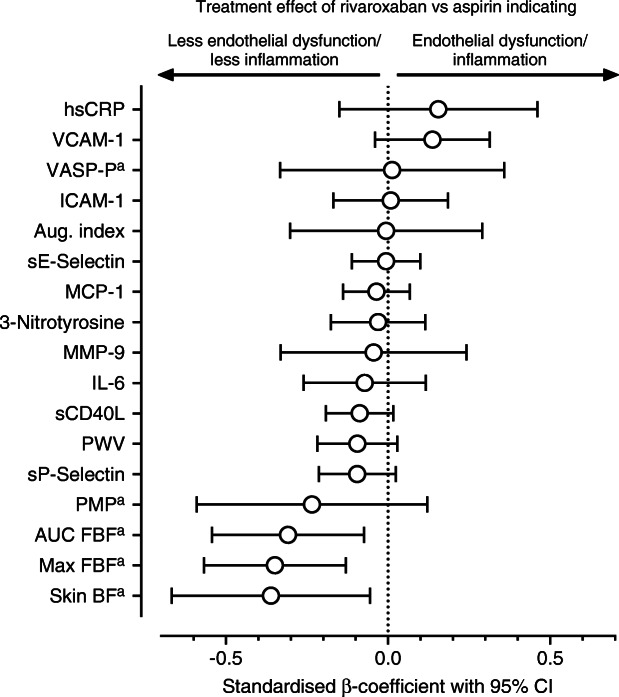
Standardised β-coefficients of the factor ‘treatment’ in increasing order for all secondary outcome variables. β-Coefficients below zero indicate improvement of endothelial function or less inflammation. AUC FBF, area under the FBF curve; aug. index, augmentation index; max FBF, maximal FBF; skin BF, skin blood flow; VASP-P, phosphorylated VASP. ^a^β-Coefficients displayed inverted to improve readability of the figure

Figure 3 shows the standardised β-coefficients of the factor ‘treatment’ for all outcome variables which confirm the inferential results displayed in Table 2: rivaroxaban vs aspirin treatment was associated with an improvement of most measures for endothelial function, i.e. FBF_max_, AUC of FBF, skin blood flow, sP-Selectin or PMPs (which stimulate endothelial repair), whereas biomarkers of inflammation (hsCRP) were less affected or even improved by aspirin. A subgroup analysis between male and female participants did not reveal statistically significant sex differences of the treatment effects, albeit there was some heterogeneity (ESM Fig. 2).

For safety reasons we also assessed bleeding events: one clinically relevant non-major bleeding and two minor bleedings but no major bleeding (as defined according to the criteria of the International Society on Thrombosis and Haemostasis) occurred with aspirin treatment, and one major bleeding, 11 clinically relevant non-major bleedings and eight minor bleedings with rivaroxaban treatment. A complete listing of bleeding events is outlined in ESM Table 1. Univariate negative binominal regression analysis, whereby the number of bleeds per participant was modelled as a linear combination of treatment and the changes of different variables of the coagulation pathway, revealed a significant impact of assignment to rivaroxaban treatment for bleeding risk (*p* < 0.01). Change of different variables of the coagulation pathway between end of treatment and baseline, e.g. prothrombin time, activated partial thromboplastin time and PTF 1+2, did not independently predict the risk of bleeding. Further serious adverse events were similar between treatment groups (see ESM Tables 2, 3).

## Discussion

Our study demonstrated that treatment with the direct factor Xa inhibitor rivaroxaban in individuals with type 2 diabetes mellitus and subclinical inflammation resulted in an improvement of FBF and microvascular skin blood flow vs aspirin. In addition, we found a reduction of sP-Selectin plasma levels with rivaroxaban and a reduction of hsCRP levels with aspirin treatment compared with the corresponding baseline values. Further serum biomarkers of endothelial function (sE-Selectin, ICAM-1, VCAM-1, 3-nitrotyrosine) or inflammation (MCP-1, IL-6, MMP-9, sCD40L) and PWV were not different between treatments. Measures of platelet activation (VASP Ser_157_ phosphorylation and number of PMPs) revealed no significant differences between treatments but HUVEC proliferation in vitro was significantly stimulated by PMPs from rivaroxaban-treated participants vs aspirin.

Endothelial dysfunction is a proatherogenic state that is characterised by diminished release of endothelium-dependent vasodilators and enhanced expression of cell adhesion molecules. An improvement of endothelial function with rivaroxaban has already been demonstrated in animal models of diabetes in vitro but not in human studies in vivo so far [26]. Our tests of endothelium-dependent vasodilatation using forearm occlusion plethysmography and laser-Doppler fluxmetry were non-invasive and well established in clinical settings [27] and demonstrate the capability of endothelial cells to release vasodilators such as NO, prostaglandins or hyperpolarising factors. Furthermore, testing of FBF was a reliable predictor of subsequent cardiovascular events in previous studies [28]. We did not differentiate between NO-dependent and NO-independent vasodilatation because an intra-arterial infusion of specific inhibitors of NO was not possible due to ethical reasons.

As demonstrated in Fig. 3, rivaroxaban treatment was associated with improved endothelium-dependent vasodilation, reduced plasma level of sP-Selectin and increased number of PMPs. All these measures indicate that rivaroxaban treatment was associated with an improvement of endothelial function although not all endothelial biomarkers were equally affected. A possible explanation for these favourable effects is the bidirectional association between inflammation, hypercoagulation and endothelial dysfunction. Inflammation can activate the coagulation cascade [29] and factor Xa or thrombin can initiate production of proinflammatory interleukins and cell adhesion molecules, endothelial dysfunction and platelet activation via PAR [10]. On the other hand, thrombin can also control coagulation by activation of protein C, a potent anticoagulant and anti-inflammatory molecule [30]. In our study, rivaroxaban treatment resulted in an inhibition of factor Xa and reduced thrombin generation (indirectly assessed by PTF 1+2) without a change in protein C activity (Table 1). Furthermore, we found a significant inverse correlation between maximal FBF and PTF 1+2 in the univariate analysis (*r* = −0.16, *p* = 0.04). Therefore, it is possible that inhibition of factor Xa or thrombin contributed to the improvement of endothelial function. In addition, animal studies have demonstrated improvement of endothelial function with factor Xa and thrombin inhibition in vitro [26, 31], mainly by improving NO-dependent vasodilatation. On the other hand, there is growing evidence that rivaroxaban can directly affect endothelial cells and platelets beyond its anti-coagulatory actions. This is indicated by the reduction of sP-Selectin level and the increase of PMPs in the present study. A reduction of Selectin-mRNA in HUVECs has also been demonstrated by pre-treatment with rivaroxaban in vitro [32]. P-Selectin mediates the binding of platelets and mononuclear cells to the endothelium and hence promotes plaque formation, and reduced levels of P-Selectin might therefore be associated with endothelial protection [10]. The number of PMPs is increased in metabolic diseases, e.g. type 2 diabetes and the metabolic syndrome, and might serve as a risk marker for atherosclerosis in cross-sectional studies [33], but it has also been demonstrated that PMPs are involved in the regulation of endothelial repair [23]. The latter mechanism was supported by our finding of increased HUVEC proliferation in cell culture with PMPs from rivaroxaban-treated participants (ESM Fig. 1). Furthermore, studies have demonstrated that rivaroxaban directly stimulated HUVEC growth in culture and endothelial repair after limb ischaemia in animal models [34, 35]. However, there is some heterogeneity for endothelial biomarkers or measures of platelet activation in our trial (Fig. 3). We did not find any effect on VASP Ser_157_ phosphorylation, VCAM-1, ICAM-1 or 3-nitrotyrosine. This is in contrast to recently published data in animal models [36] or cell culture [32] which demonstrated a reduction of circulating VCAM-1 and ICAM-1 levels or mRNA; however, these studies used lipopolysaccharide or thrombin for acute induction of these adhesion molecules to a level tenfold or 100-fold above baseline, which is far beyond a physiological level in humans. In addition, there is a remarkable endothelial cell heterogeneity even within an organ system and the degree of endothelial dysfunction can vary between different vascular regions depending on the microenvironment [37]. Therefore, it is possible that due to dilution effects, soluble markers of endothelial dysfunction in vivo are not as sensitive as provocative tests to detect small changes. This would partly explain our heterogeneous findings. The lack of an effect on VASP Ser_157_ phosphorylation did not exclude a possible VASP activation because of further phosphorylation sites. Furthermore, there was some heterogeneity between male and female participants regarding this variable as well as ICAM-1 or 3-nitrotyrosine (ESM Fig. 2). It is therefore possible that sex differences, albeit not statistically significant in the present study, could have an influence on the endothelial or platelet response to rivaroxaban. There was only a moderate treatment effect on PWV. PWV rather indicates structural abnormalities of the vessel wall than early endothelial dysfunction. Therefore, we can assume that 20 weeks of rivaroxaban treatment did not significantly improve stiffness of large conductance arteries. In contrast to our findings, a short-term investigation over 3 months demonstrated an improvement of arterial stiffness with rivaroxaban treatment compared with warfarin in individuals with non-valvular atrial fibrillation; however, this study mainly represented a worsening of arterial stiffness with warfarin rather than an improvement with rivaroxaban [12].

We found only a few treatment effects on inflammatory biomarkers. Aspirin significantly reduced hsCRP as reported in previous large studies [38] but rivaroxaban had no significant effect in comparison with the corresponding baseline values (Table 2). A treatment effect vs aspirin is difficult to obtain since aspirin itself is a potent anti-inflammatory drug. This finding is in contrast to animal studies which demonstrated a significant reduction of MCP-1 and IL-6 plasma levels with rivaroxaban treatment [36]; however, most of these studies were of short duration and induced acute inflammatory responses. One recently published trial of 918 individuals with atrial fibrillation reported a small but statistically significant 10% reduction of hsCRP and IL-6 plasma levels after 42 days of rivaroxaban treatment [39]; however, all patients received electrical cardioversion which could have influenced the results. Although there is growing evidence for an anti-inflammatory effect of rivaroxaban, we were unable to detect it in our study using established biomarkers. This lack of findings could be partly related to the lack of statistical power for detection of small changes of biomarkers.

A major disadvantage of direct factor Xa inhibition is the higher risk of bleeding compared with antiplatelet therapy alone. The risk increases with higher doses of these drugs or in combination with antiplatelet agents. Interestingly, there is no inverse relationship between bleeding risk and reduction of MACE [40]. Therefore, it is important to identify vulnerable patients and to adapt the dosage of direct factor Xa inhibitors to achieve sufficient inhibition of thrombin generation without an excessive increase of bleeding risk.

Our study has some limitations, i.e. the open-label design which potentially biased the results and the lack of a combined treatment arm which prevented the analysis of possible interactions between aspirin and rivaroxaban. However, since this was a mechanistic study, we focused on detection of different effects on endothelial function between aspirin and rivaroxaban treatment. The study also has some strengths: medical staff and investigators who performed laboratory measurements, assessed vascular function and analysed the results were blinded regarding the treatment assignment; the number of participants was acceptable; and we used different methods for assessment of endothelial function.

Taken together, our findings indicate that one possible mechanistic explanation for the favourable clinical effects of the direct factor Xa inhibitor rivaroxaban is the improvement of endothelial function. Further trials of longer duration are needed to clarify whether inhibition of factor Xa can reduce cardiovascular outcomes with an acceptable risk of bleeding in an early stage of atherosclerosis.

## Supplementary information

ESM (PDF 316 kb)

## Data Availability

The clinical study report is available at ClinicalTrials.gov (registration no. NCT02164578). Original data are available on request from the corresponding author.

## Abbreviations

FBF: Forearm blood flow
hsCrP: High-sensitivity C-reactive protein
ICAM-1: Intercellular adhesion molecule 1
MACE: Major adverse cardiovascular events
maxBF: Maximal post-ischaemic blood flow
MCP-1: Monocyte chemoattractant protein 1
MMP-9: Matrix metallopeptidase 9
NO: Nitric oxide
PAR: Protease-activated receptors
pBF: Pre-ischaemic blood flow
PE: Phycoerythrin
PMP: Platelet-derived microparticle
PTF: Prothrombin fragment
PWV: Pulse wave velocity
sCD40L: Soluble CD40 ligand
sE-Selectin: Soluble E-Selectin
sP-Selectin: Soluble P-Selectin
VASP: Vasodilator-stimulated phosphoprotein
VCAM-1: Vascular cell adhesion molecule 1
WST-1: 4-[3-(4-iodophenyl)-2-(4-nitrophenyl)-2H-5-tetrazolio]-1,3-benzene disulfonate
