# Supplementary material for: Rivaroxaban compared with low-dose aspirin in individuals with type 2 diabetes and high cardiovascular risk: a randomised trial to assess effects on endothelial function, platelet activation and vascular biomarkers
Source: Diabetologia. 2021 Sep 8;64(12):2701–12. doi: 10.1007/s00125-021-05562-9 (PMC8563606; doi:10.1007/s00125-021-05562-9)
Supplement: Supplementary file 1 — (PDF 316 kb) [file 125_2021_5562_MOESM1_ESM.pdf]

Supplementary Appendix

Content | Page

1. ESM Methods
   - List of investigators and study sites 1
   - Randomisation sequence generation and time plan of recruitment 1
   - Complete list of inclusion and exclusion criteria 1
   - Detailed description of statistical analyses 3
2. ESM tables 5
3. ESM figures 7

1. ESM Methods

List of investigators and study sites:

Ingo Weigmann, Elena Henkel: GWT-TU-Dresden GmbH, Centre for Clinical studies, Dresden, Germany

Heidrun Mehling: Studienzentrum Charite, Berlin-Buch, Germany

Christoph Axthelm, Kristin Kreutzmann: Cardiologicum Pirna, Germany

Frank Schaper: Diabetesschwerpunktpraxis, Dresden, Germany

Randomization sequence generation and time plan of recruitment:

All eligible participants were randomized at baseline visit V1 using a central block randomization process based at the sponsor's site (blocks of 4, each block containing 2 allocations per treatment at random order). The study site requesting randomization of a participant sent a form containing site ID and screening number to the sponsor. In return, the site received a randomization form containing the randomization number and allocation of the patient to the respective treatment arm by fax or email within 30 min.

First patient first visit (FPFV)	14 April 2015
Last patient last visit (LPLV)	12 Dec 2018
Clean database	14 Feb 2019

Complete list of inclusion and exclusion criteria:

Inclusion criteria

Subjects had to fulfill all of the following criteria to be included in the study:

- Type 2 diabetes duration between 2 and 20 years
- Two or more components of metabolic syndrome:

    - High-density lipoprotein cholesterol < 1.0 mmol/L (in men) or < 1.3 mmol/L (in women)
    - Elevated triglycerides (> 1.7 mmol/L)
    - Elevated blood pressure (> 130 mmHg systolic and/or > 85 mmHg diastolic or antihypertensive treatment)
    - Elevated waist circumference (> 102 cm in males, > 85 cm in females)
- OR at least one of the following
    - Carotid ultrasound showing an intima media thickness > 1 mm and plaque of carotid artery or
    - Left ventricular hypertrophy or
    - Increased urine albumin to creatinine ratio (> 2.6 mg/mmol) in the absence of other renal diseases than diabetic nephropathy
- Increased high-sensitivity C-reactive protein (> 2 mg/l but < 10 mg/l) at or within 6 months prior to screening and/or increased plasminogen activator inhibitor 1 (> 15 ng/ml) at or within 6 months prior to screening
- Age 40 – 75 years at the first screening visit
- Stable treatment with statins (if tolerated)
- The ICF must have been signed before any study specific tests or procedures were done
- Ability to understand and follow study-related instructions

Exclusion criteria
Subjects were to be excluded from the study if they displayed any of the following criteria:

- Major cardiovascular event with need for oral anticoagulation or platelet inhibitor therapy or acute coronary syndrome < 12 month before study entry
- Sustained uncontrolled hypertension: systolic blood pressure > 180 mmHg or diastolic blood pressure > 100 mmHg
- Known hypersensitivity to the active substance or to any of the excipients
- Active clinically significant bleeding
- Lesion or condition considered to be a significant risk for major bleeding including the following
    - Active internal bleeding
    - Platelet count < 100.000/µL at the screening visit
    - Current or recent gastrointestinal ulceration
    - Presence of malignant neoplasm at high risk of bleeding
    - Recent brain or spinal injury, recent brain, spinal or ophthalmic surgery
    - Recent intracranial hemorrhage
    - Known or suspected esophageal varices, arteriovenous malformations, vascular aneurysms or major intraspinal or intracerebral vascular abnormalities
- Concomitant treatment with any other anticoagulants e.g. unfractionated heparin (UFH), low molecular weight heparins (enoxaparin, dalteparin, etc.), heparin derivatives (fondaparinux, etc.), oral anticoagulants (warfarin, dabigatran etexilate, apixaban, etc.) except under the circumstances of switching therapy to or from rivaroxaban or when UFH was given at doses necessary to maintain an open central venous or arterial catheter

- Concomitant treatment of acute coronary syndrome with antiplatelet therapy in patients with a prior stroke or a transient ischemic attack (TIA)
- Hepatic disease associated with coagulopathy and clinically relevant bleeding risk including cirrhotic patients with Child Pugh B and C
- Chronic renal failure with estimated glomerular filtration rate < 15 ml/min (MDRD formula)
- As with other antithrombotics, rivaroxaban was not recommended in patients with an increased bleeding risk such as:
    - Congenital or acquired bleeding disorders
    - Uncontrolled severe arterial hypertension
    - Active ulcerative gastrointestinal disease
    - Vascular retinopathy
    - Bronchiectasis or history of pulmonary bleeding
- Known human immunodeficiency virus infection at time of screening
- Pregnant or breast-feeding women and women without adequate method of contraception
- Subject was in custody by order of an authority or a court of law
- Exclusion periods from other studies or simultaneous participation in other clinical studies
- Previous assignment to treatment during this study
- Scheduled (elective) surgery after signing the ICF
- Close affiliation with the investigator (e.g. a close relative) or persons working at the study site
- Subject was an employee of GWT-TUD GmbH or Bayer Vital GmbH
- Criteria which in the opinion of the investigator precluded participation for scientific reasons, for reasons of compliance, or for reasons of the subject’s safety

Detailed description of statistical analysis

Sample Size calculation:

The primary variable was change of FBF with venous occlusion plethysmography(ΔFBF) after forearm ischemia after 20 weeks treatment. The sample size calculation was based on the following assumptions:

$$H0: \Delta\ FBF_{Riva} \leq \Delta\ FBF_{ASA}$$
$$H1: \Delta\ FBF_{Riva} > \Delta\ FBF_{ASA}$$

- Type I error: $\alpha = 0.05$, one-tailed t-test for independent samples
- Power: 1-ß = 0.90
- Estimated dropout rate: 25 %
- Minimum of expected clinically relevant difference between the groups in changes of maximal FBF (ΔFBF) = 1.2 ml/100ml with an estimated standard deviation (SD) of σ = 2.4 ml/100ml

Sample size was calculated with N = 188 participants ($N_{1/2}$ = 94 patients per arm) to reach a valid number of 141 participants for per protocol analysis. Recruitment was stopped after randomization of 179 participants due to the lower than expected number of drop outs.

Descriptive or summary statistics:

All continuous variables were summarized by treatment group using the following statistics: *n* (non-missing sample size), number of missing values (Missing), arithmetic mean (Mean), standard deviation (SD), median (Median), maximum (Max) and minimum (Min).

Tables for original (untransformed) data were provided as well as tables for differences from baseline by visit. Boxplots were presented for illustration.

For nominal or categorical data, frequencies and percentages (based on the non-missing sample size) were tabulated for each category.

Hypothesis testing:

Unless otherwise specified all hypotheses were tested at a 5% level of significance against two-sided alternative hypotheses.

Continuous variables were analyzed by the following test procedures:

Firstly, the assumption of normally distributed data was checked with Shapiro-Wilk test by treatment group. If the hypothesis of normally distributed data was not rejected for both treatment groups, treatments were compared by an ANCOVA procedure which includes “treatment” as the main factor and “baseline value” as covariate. T-Tests for independent samples were used to compare groups at baseline.

In the event that the normality assumption was not warranted, data are log-transformed and normality assumption of the transformed data was checked by Shapiro-Wilk test. In case of non-rejection of the hypothesis of normally distributed data for both treatment groups, ANCOVA procedure and t-tests as described above were performed on the basis of log-transformed data.

Point estimates and 95% CI of the difference in (adjusted) mean change from baseline were provided if data are evaluated on basis of an ANCOVA procedure.

Otherwise Mann-Whitney-U test on the basis of original data was applied for comparison of groups at baseline. Mann-Whitney-U test on the basis of intra-individual differences to baseline was applied for treatment comparison per visits after baseline. Wilcoxon´s signed rank test was applied to evaluate changes from baseline per treatment group.

Categorical variables were analyzed using χ^2- test to compare proportions between treatment groups. Additionally, for each category, the proportions of treatment groups were compared using an (asymptotic) z test if applicable. Significant differences in proportions will be flagged, p-values for the z-test will not be shown.

Missing values were not be imputed.

2. ESM tables

ESM table 1. List of bleeding events (according to International Society on Thrombosis and Hemostasis), CRNM: critical non major

		Rivaroxaban		Aspirin		Total	
		n	%	*n*	%	N	%
Number of Events	Major	1		0		1	
	CRNM	11		1		12	
	Minor	8		2		10	
	Total Bleedings	20		3		23	
Number of Patients	Major	1	1.1	0	0.0	1	0.6
	CRNM	7	7.9	1	1.1	8	4.5
	Minor	3	3.4	2	2.2	5	2.8
	Total Bleedings	11	12.4	3	3.3	14	7.8

ESM table 2. Summary of adverse events (AE) and severe adverse events (SAE)

	Rivaroxaban *n* = 89 (100%)	Aspirin *n* = 90 (100%)	Total *N* = 179 (100%)
Number (%) of subjects with at least one AE	63 (70.8%)	62 (68.9%)	125 (69.8%)
Number of AEs	179	148	327
Number of study drug related AEs	53 (29.6%)	29 (19.6%)	82 (25.1%)
Number (%) of subjects with at least one SAE	4 (4.5%)	6 (6.7%)	10 (5.6%)
Number of SAEs	11	12	23
Number of study drug related SAE	0	0	0

ESM table 3. Listing of severe adverse events (SAE)

	Adverse event term - SAE	Rivaroxaban	Aspirin
Gastrointestinal disorders			
	Gastrointestinal hemorrhage	1	0
	Nausea	0	1
	Vomiting	0	1
General disorders and administration site conditions			
	Chest pain	0	1
Hepatobiliary disorders			
	Bile duct stone	0	1
Infections and infestations			
	Diverticulitis	0	1
	Erysipelas	0	1
	Escherichia sepsis	2	0
Injury, poisoning and procedural complications			
	Foot fracture	0	1
Musculoskeletal and connective tissue disorders			
	Intervertebral disc protrusion	0	1
	Osteoarthritis	1	0
Neoplasms benign, malignant and unspecified (incl cysts and polyps)			
	Leiomyoma	1	0
Nervous system disorders			
	Cerebrovascular accident	0	1
	Syncope	1	0
Reproductive system and breast disorders			
	Ovarian cyst	1	0
Respiratory, thoracic and mediastinal disorders			
	Asthma	1	0
Surgical and medical procedures			
	Hysterectomy	1	0
	Joint arthroplasty	1	0
Vascular disorders			
	Hypertension	1	0
	Hypertensive crisis	0	3
Total		11	12

3. ESM Figures

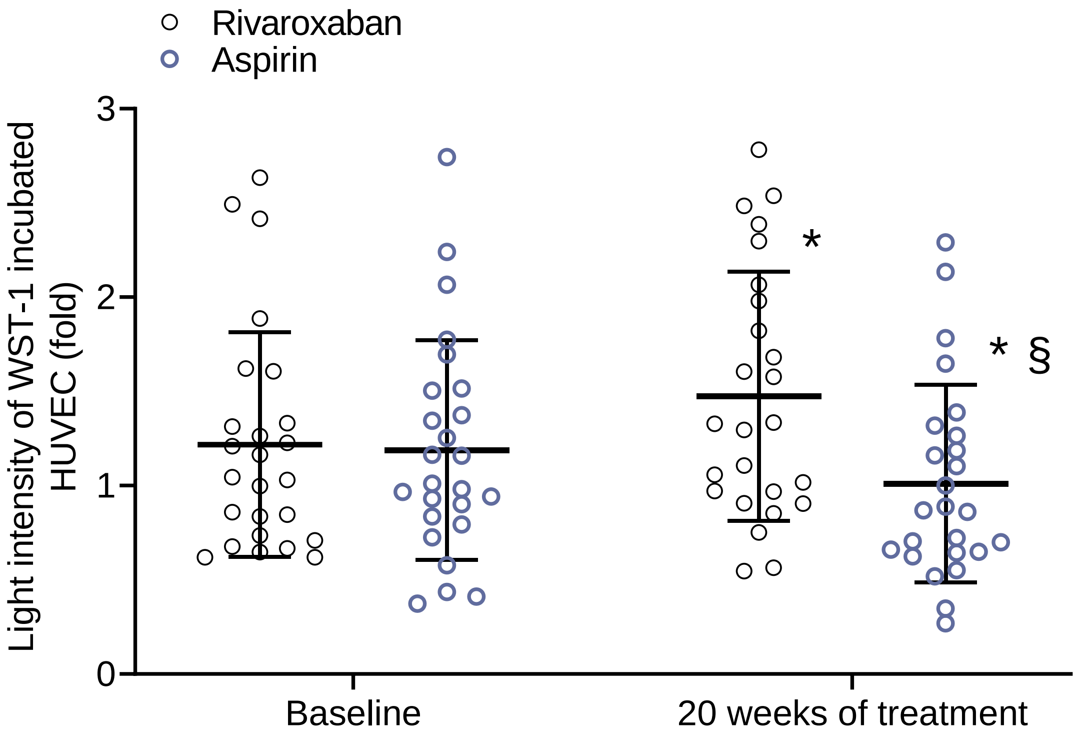

ESM Figure 1: Proliferation of HUVEC after incubation with PMP of treated patients. *p<0.05 vs baseline, § p<0.05 vs rivaroxaban

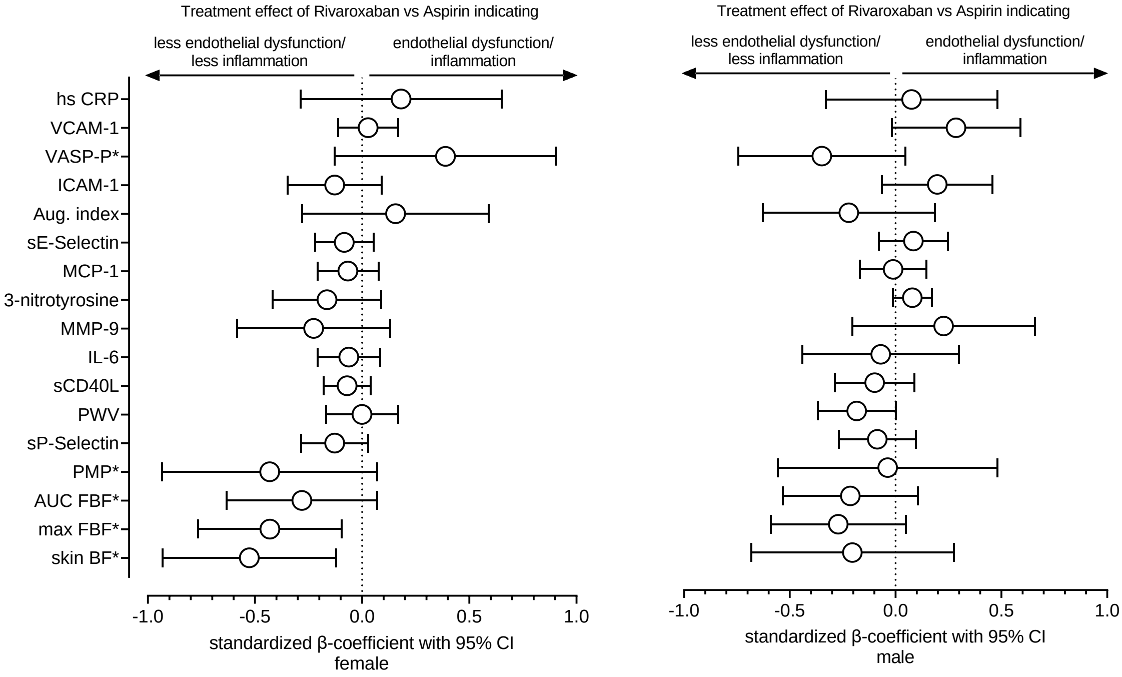

ESM Figure 2: Standardized β-coefficients of the factor ´treatment` separated by sex. β-coefficients below zero indicate improvement of endothelial function or less inflammation. Aug. index, augmentation index; VASP-P, phosphorylated VASP; skin BF, skin blood flow; max FBF, maximal FBF; AUC FBF, area under the FBF curve. *β-coefficients displayed inverted to improve readability of the figure.
